# CNV Analysis through Exome Sequencing Reveals a Large Duplication Involved in Sex Reversal, Neurodevelopmental Delay, Epilepsy and Optic Atrophy

**DOI:** 10.3390/genes15070901

**Published:** 2024-07-10

**Authors:** Cybel Mehawej, Joy El Maalouf, Mohamad Abdelkhalik, Peter Mahfouz, Eliane Chouery, Andre Megarbane

**Affiliations:** 1 Department of Human Genetics, Gilbert and Rose-Marie Chagoury School of Medicine, Lebanese American University, Byblos P.O. Box 36, Lebanon; cybel.mehawej@lau.edu.lb (C.M.); eliane.choueiry01@lau.edu.lb (E.C.); 2 Gilbert and Rose-Marie Chagoury School of Medicine, Lebanese American University, Byblos P.O. Box 36, Lebanon; joy.maalouf@lau.edu (J.E.M.); peter.mahfouz@lau.edu (P.M.); 3 Institut Jérôme Lejeune, 75015 Paris, France

**Keywords:** X chromosome duplication, sex reversal, copy number variant, exome sequencing, genetic counseling

## Abstract

Background: Duplications on the short arm of chromosome X, including the gene *NR0B1*, have been associated with gonadal dysgenesis and with male to female sex reversal. Additional clinical manifestations can be observed in the affected patients, depending on the duplicated genomic region. Here we report one of the largest duplications on chromosome X, in a Lebanese patient, and we provide the first comprehensive review of duplications in this genomic region. Case Presentation: A 2-year-old female patient born to non-consanguineous Lebanese parents, with a family history of one miscarriage, is included in this study. The patient presents with sex reversal, dysmorphic features, optic atrophy, epilepsy, psychomotor and neurodevelopmental delay. Single nucleotide variants and copy number variants analysis were carried out on the patient through exome sequencing (ES). This showed an increased coverage of a genomic region of around 23.6 Mb on chromosome Xp22.31-p21.2 (g.7137718-30739112) in the patient, suggestive of a large duplication encompassing more than 60 genes, including the *NR0B1* gene involved in sex reversal. A karyotype analysis confirmed sex reversal in the proband presenting with the duplication, and revealed a balanced translocation between the short arms of chromosomes X and 14:46, X, t(X;14) (p11;p11) in her/his mother. Conclusions: This case highlights the added value of CNV analysis from ES data in the genetic diagnosis of patients. It also underscores the challenges encountered in announcing unsolicited incidental findings to the family.

## 1. Introduction

Male sex reversal, occurring in 1:20,000–100,000 of males, is a condition where the sexual phenotype of an individual is in the opposite direction to his genetic sex [1].

This is due to an alteration of an organized cascade of regulatory interactions between specific transcription factors acting on the bipotential undifferentiated gonads and leading to their segregation into either the ovary or the testis. In mammals, the Y chromosome has a potent testis-determining effect on the indifferent stage, leading to male gonad development. Located on the Y chromosome is the sex determining gene *SRY* (which stands for sex-determining region Y gene) [2]. Male sex determination is governed sequentially by sex-determining region Y (SRY) and related SRY-box 9 (SOX9) transcription factors [3]. During male embryogenesis, and as early as day 41, *SRY* is expressed and leads to the differentiation, supporting cell precursors into Sertoli cells rather than follicle cells [2]. SRY then synergizes with steroidogenic factor1 (SF1, also known as nuclear receptor subfamily 5, group A, member 1), and binds to the testis-specific enhancer of the *SOX9* gene (sry-related HMG box-9) to induce its expression [4]. *SOX9* is another definitive testis differentiation gene. It acts as a transcriptional repressor and activator as well. Indeed, after its expression reaches a threshold, SOX9 downregulates the *SRY* gene, while activating downstream signaling pathways such as FGF9 and PGD2, repressing ovary-determining genes, and potentially activating *SOX8* [5]. The latter then acts redundantly with *SOX9*. Their expression is maintained in Sertoli cells throughout life and they are important for maintaining adult testis [6,7].

Interference during embryogenesis in the tightly regulated sex determination process can be due to dysregulation in the expression of some key genes. This results in a failure to either suppress the opposite pathway (ovarian development) or to maintain the induced pathway (testis development).

Consequently, gonadal dysgenesis—in other words, reduced or totally absent gonads—may occur [8]. To date, around 11 genes with different inheritance modes have been linked to male sex reversal. Duplications of the short arm of the X chromosome have also been reported to be involved in sex reversal and in female/ambiguous external genitalia [9,10,11,12,13,14,15,16,17,18,19,20]. These duplications affect the DSS (dosage-sensitive sex reversal) region that is mapped to Xp21.1-Xp22.1 range [21]. Interestingly, this region includes the *NR0B1* (nuclear receptor subfamily 0, group B, member 1) gene, also known as *DAX1* (dosage-sensitive sex reversal-adrenal hypoplasia congenita critical region on the X chromosome, gene 1). NR0B1 regulates the development of the hypothalamic pituitary adrenal gonadal axis, and plays an essential role in sex determination and differentiation. It mainly functions as a transcriptional repressor, which affects various critical factors involved in sex determination. Indeed, the crucial role of DAX1 in male sex determination is carried out through several pathways, including the inhibition of the activity of SF1, which has a fundamental role in activating the SRY gene [22]. DAX1 also downregulates the expression of the SOX9 gene, which is crucial for the development of testes [23]. The dosage sensitivity of DAX1 is crucial, and excessive levels of DAX1 can antagonize the action of SRY, which leads to male-to-female sex reversal [21]. In other words, an XY person with a duplication involving DAX1 leads to the excessive inhibition of SRY, leading to a female phenotype. This relationship between DAX1 and the other sex-determining genes highlights the importance of the tight regulation needed for normal sex development and preventing disorders of sex development (DSDs), including sex reversal as well as conditions with both ovarian and testicular tissues (ovotesticular DSD) [21].

Mutations in the NR0B1 gene can lead to several genetic conditions due to dysfunction in adrenal and reproductive functions. While point mutations and deletions affecting this gene cause congenital adrenal hypoplasia and hypogonadotropic hypogonadism [24], duplications including the *NR0B1* gene have been detected in XY individuals presenting phenotypically as females [21,22,23]. In the case of chromosomal abnormalities altering the gene, additional clinical manifestations can be observed in the patient depending on the genomic region that is affected [25].

Here, we present a 23.6 MB duplication on chromosome X in a 2-year-old patient with male sex reversal, dysmorphic features, optic atrophy, epilepsy, psychomotor and neurodevelopmental delay. A comprehensive review of the literature reporting duplications between Xp22.31-p22.12 (chrX:7,137,718–30,739,112) and their associated symptoms is also included.

## 2. Material and Methods

### 2.1. Patient

The proband, a 2-year-old female patient with dysmorphic features, optic atrophy, epilepsy, psychomotor and neurodevelopmental delay, was referred to our department for genetic testing and family counseling. A work-up, comprising a thorough clinical evaluation and molecular analyses, was performed. Written informed consent from the patient’s parents was obtained. 

### 2.2. Isolation of Genomic DNA

A peripheral blood sample was collected from the patient for genetic studies. DNA was extracted from leucocytes by standard salt-precipitation methods [26]. 

### 2.3. Exome Sequencing (ES)

ES was carried out in the patient. The exome was captured and enriched using the solution Agilent SureSelect Human All Exon kit version 5.0, and samples were then multiplexed and subjected to sequencing on an Illumina HiSeq 2500 PE100-125. Reads files (FASTQ) were generated from the sequencing platform via the manufacturer’s proprietary software. Reads were aligned to the hg19/b37 reference genome using the Burrows–Wheeler Aligner (BWA) package version 0.7.11 [27]. Variant calling was subsequently performed using the Genome Analysis Tool Kit (GATK) version 3.3 [28]. Variants were called using high stringency settings and annotated with VarAFT software 1.61 [29], containing information from dbSNP147 and the Genome Aggregation database (gnomAD, http://gnomad.broadinstitute.org, accessed on 1 May 2023). Only the nonsynonymous coding and splicing variants found in the patient were considered. Variant filtering was performed according to the mode of transmission of the disease in the family, the frequency of the variant in the gnomAD database (<0.01% and <50 heterozygous carriers or <5 homo-/hemizygous carriers), and in our in-house database (<1 homozygous occurrence).

### 2.4. CNV Analysis—ExomeDepth

ES data were analyzed for the detection of copy number variants (CNVs), as reported earlier [30]. Briefly, the coverage of each sequencing amplicon is analyzed, and its sequencing depth is compared to that of all the samples processed on the same sequencing run. Several normalization steps are then performed to compute a score for each amplicon. A theoretical score of 1 is the normal case, suggesting that the amplicon was amplified similarly to that in other samples, while low (<0.5) or high (>1.5) values, respectively, reveal deletions or duplications. Cov’Cop and CovCopCan were used with the default settings, with all options active, and we defined a minimum threshold of three successive amplicons on the same chromosome to highlight a CNV. 

### 2.5. Blood Karyotyping

Peripheral blood was withdrawn from the proband and her/his parents, and blood karyotyping was performed, following standard protocols based on R banding. Briefly, lymphocyte cultures were initiated from the peripheral blood samples withdrawn from the tested individuals. Colchicine was added to stop cells in metaphase, followed by hypotonic treatment and fixation to prepare metaphase spreads. Slides were then treated with trypsin and stained using Giemsa, enabling the visualization of R bands under a microscope. Chromosomal abnormalities were identified and classified according to the International System for Human Cytogenetic Nomenclature (ISCN) guidelines. An analysis was conducted using a light microscope equipped with appropriate software for image capture and karyotype interpretation (Metasystem/Zeiss Axio-Imager Z2 and Metafer Scanning System, River’s Edge Drive, Medford, OR, USA).

## 3. Results

### 3.1. Case Presentation

The patient is the third child born to a non-consanguineous Lebanese couple. The parents, who were both healthy, have an unaffected boy and have had a baby girl with multiple malformations, lost at 8 months of pregnancy without being genetically investigated. The patient was born at term through spontaneous vaginal delivery. She/he had normal female external genitalia. At birth, her/his weight was 2800 g and her/his length was 50 cm. During pregnancy, growth retardation was noted, but no known toxic exposures nor unusual events were reported. 

By the age of 1 month, parents noted absence of eye contact, and strabismus. Ophthalmic examination revealed optic nerve dysgenesis and a macular coloboma on the right side. A brain MRI was performed, showing bilateral optic nerve atrophy. At 6 months old, following multiple urinary tract infections, the child was diagnosed with vesicoureteral reflux and underwent surgery two years later. Additionally, since she/he was 6 months old, the patient experienced epileptic spasms, confirmed by EEG, indicating a diagnosis of West syndrome, for which she/he was treated with adrenocorticotropic hormone.

The patient was first examined by us at the age of 2 years. Her/his height was 75.5 cm; weight, 7500 g; and head circumference, 45 cm (all below the 3rd percentile). A physical examination revealed a high-arched palate, telecanthus, strabismus, a broad nasal bridge, joint hyperlaxity, a single palmar crease on the left hand, long fingers, and muscle hypotrophy. She/he displayed severe developmental delay, responding only by smiling to her/his parents’ voices, and was unable to sit unaided. She/he also exhibited marked hypotonia. Feeding difficulties were reported, along with gastroesophageal reflux. Auscultation detected a systolic ejection murmur. Echocardiography revealed the presence of an ostium secundum atrial septal defect. A brain MRI showed abnormal high T2W/FLAIR signal intensity in the periventricular white matter around the posterior horn. Laboratory results indicated elevated FSH and LH levels.

### 3.2. Genetic Results

Exome sequencing (ES) carried out on the patient allowed for the detection of 100,237 genetic variants. The detected ES variants were filtered for only the protein-altering variants, including canonical splice-site variants, whose frequency of occurrence in public repertoires and our in-house database is lower than 1% (as detailed above). ES analysis did not detect any candidate variant that may explain the clinical manifestations in the patient. However, surprisingly, it revealed the presence and full coverage of chromosome Y. CNV analysis using ES coverage data was then performed, showing increased coverage of a genomic region of around 23.6 MB on chromosome Xp22.31-p21.2 (g.7137718–30739112), suggestive of a duplication that was never reported in the Database of Genomic Variants (DGV). This region comprises a total of 143 genes, of which 24 are dosage-sensitive (Table 1, updated from Genescout November 2023). Among these, 11 are triplosensitive (Table 1), including the *NR0B1* gene involved in 46XY sex reversal 2, which is also dosage-sensitive.

**Figure 1:**
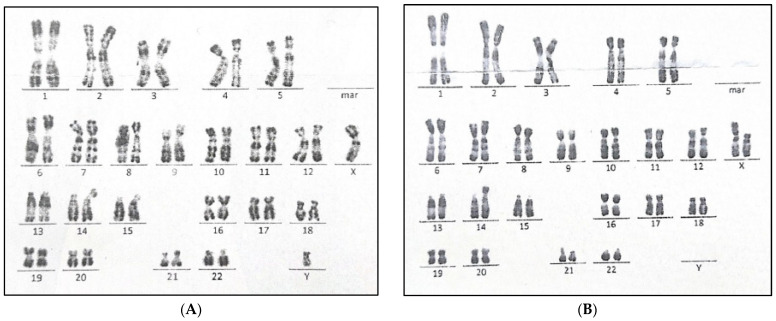
Blood karyotyping in the family showing (**A**) a male pattern with an additional chromosomal fragment of a maternal origin on the short arm of chromosome 14—46,XY,matder(14;X)(p11), in the propositus; and (**B**) a balanced translocation between the short arms of chromosomes X and 14 in the mother—46,X,t(X;14)(p11;p11).

Blood karyotyping was then carried out on the propositus, showing a male pattern with an additional chromosomal fragment of an unknown origin on the short arm of chromosome 14 (Figure 1A). A karyotype analysis, performed on both parents, revealed the presence of an apparently balanced translocation between the short arms of chromosomes X and 14 in the mother—46,X,t(X;14)(p11;p11) (Figure 1B). The chromosomal formula of the propositus was then as follows: 46,XY,matder(14;X)(p11). Following this result, genetic counseling addressing this sensitive matter was provided to the family, in the presence of the psychologist who was in charge of handling the appropriate follow-up.

## 4. Discussion

Here, we report a 23.6 Mb duplication on chromosome Xp22.31-p21.2 (g.7137718–30739112), detected by CNV analysis through ES in a 2-year-old Lebanese patient presenting with male sex reversal, dysmorphic features, optic atrophy, epilepsy, psychomotor and neurodevelopmental delay. A karyotype analysis showed that the duplication results from a balanced maternal translocation: 46,X,t(X;14)(p11;p11).

To our knowledge, this is one of the largest duplications reported in a patient with sex reversal. To date, large duplications of more than 1 Mb on the short arm of the X chromosome have been detected in several reported patients (Table 2). The clinical manifestations of the affected individuals depend on the size of the affected region and the genes included in it. Owing to the large size of the region herein described, each of the clinical manifestations presented by our patient are justified, since they have been reported in other patients (Table 2). For instance, neurological manifestations such as hypotonia have been reported in most of the duplications of the Xp22.31 region, as well as in a case with Xp21.3 duplication [31,32,33]. Several forms of epilepsy are also seen in cases with Xp duplications, notably Xp22.31, Xp22.2, Xp22.12, and Xp21.3, which are often accompanied with significant changes detectable on brain imaging [31,32,34,35,36]. Moreover, different degrees of intellectual disability and developmental delay, ranging from motor to speech delay, are found in cases with duplications across the whole region [31,32,33,34,35,37,38,39,40]. Neurobehavioral features such as autism are also widely observed in several duplications in the Xp region, while more specific psychiatric symptoms, such as psychosis and pre-psychosis, were found in Xp22.12 and 21.3, respectively [33,35]. Furthermore, some patients with a duplication in Xp22.2 presented with presbycusis [37], and multiple heart defects, including valvular defects, are noted in several patients with duplications affecting Xp22.2–21.3 [38,39]. Gastrointestinal problems, such as feeding difficulty and gastroesophageal reflux, are also found in patients with a duplication in Xp22.31 [31,32]. Musculoskeletal abnormalities, such as scoliosis and short stature, are also associated with Xp22.2–22.13 [39]. Joint laxity and hypermobility were noted in a single case in Xp22.12 [35]. Dysmorphic features are variable and present across different regions, ranging from microphthalmia, digit and limb deformities, talipes anomalies, among others. Notable facial dysmorphism features include hypertelorism, telecanthus, a long face and a wide nasal bridge [31,32,35,36,37,38,39,40].

To better correlate the clinical manifestations with the genes located in the duplicated genomic region, a thorough review of all genes was performed to identify dosage-sensitive genes, especially those that are triplosensitive (Table 1). Among these, *NR0B1*, a gene located at Xp21.2, encodes a member of the nuclear receptor superfamily that functions as a coregulatory protein, suppressing the transcriptional activity of other nuclear receptors. Alterations in the dosage of *NR0B1* lead to adrenal hypoplasia or to 46XY sex reversal [41], which is observed in the patient included in this study. 

Among the triplosensitive genes located in the duplicated region, *ARX*, *CDKL5*, *CNKSR2*, *MID1*, *PTCHD1*, *RPS6KA3*, and *SMS* are known to be involved in intellectual developmental disorders with/without epilepsy. For instance, an increased dosage of the *CDKL5* gene has been linked to a variety of symptoms, such as microcephaly, intellectual disability, limited hand skills, hypotonia, lack of eye contact, absence of speech and walking, seizures, and ataxia. The *ARX* gene also plays a role in cerebral development and patterning. An increase in *ARX* gene dosage results in a range of disorders, including developmental and epileptic encephalopathy, intellectual developmental disorder, lissencephaly, Partington syndrome, Proud syndrome, and hydranencephaly with abnormal genitalia, such as cryptorchidism and hypospadias. Our patient presents with epilepsy, and psychomotor and neurodevelopmental delay. He/she could not be assessed for his/her behavior (autism and ADHD), due to his/her severe intellectual disability. Attributing his clinical features to a particular gene is also very challenging.

Additionally, ophthalmologic problems, such as strabismus and optic nerve atrophy, were also reported in patients with duplications in this region [35,42]. *RS1*, located at Xp22.13, is an additional triplosensitive gene that is essential for the adhesion and interactions between cells in the retina. An increase in *RS1* gene dosage causes retinoschisis, a condition characterized by the separation of retinal layers. This leads to optic atrophy and impaired vision, which is consistent with the symptoms observed in our patient.

On the other hand, *ANOS1* has been linked to Kallmann syndrome, which is characterized by a delayed or absent puberty and impaired olfaction. An assessment of the absence of puberty is not possible due to the young age of the patient. Furthermore, a clinical evaluation for olfactory impairment could not be performed. Although some of the dosage-sensitive genes within the duplicated region were linked to specific diseases in our patient, other diseases associated with dosage-sensitive genes were not observed yet. 

Last but not least, owing to the large size of the duplication and the large number of affected genes, a regular and thorough clinical follow-up and a multidisciplinary approach are required to enable optimal care for the patient and prevent morbidity associated with the genes not yet characterized. Furthermore, counseling the family is crucial, especially given that the identification of the balanced translocation in the mother has direct consequences on the risk of a recurrence of the disease in the family. 

## 5. Conclusions

In summary, here we report a large duplication of 23.6 Mb on chromosome X, detected by CNV analysis through ES in a 2-year-old patient. This case highlights the added value of CNV analysis from ES data in the genetic diagnosis of patients. It also shows the relevance of standard techniques such as blood karyotyping in specific cases, such as in balanced translocations.

Last but not least, this case illustrates the challenges encountered in the genetic counseling of families, especially when unsolicited incidental findings are identified.

## Figures and Tables

**Table 1:** List of the dosage-sensitive genes included in the genomic region duplicated in the proband.

Cytogenetic Location	Gene	Gene Name	Gene MIM#	Function	Dosage Sensitivity	Phenotype	Phenotype MIM#
Xp22.31	*ANOS1*	anosmin 1	300836	migration of GNRH neurons to the hypothalamus	Triplosensitivity	Hypogonadotropic hypogonadism 1 with or without anosmia (Kallmann syndrome 1)	308700
Xp21.3	*ARX*	aristaless related homeobox	300382	cerebral development and patterning	Triplosensitivity	Developmental and epileptic encephalopathy 1	308350
Xp22.13	*CDKL5*	cyclin dependent kinase like 5	300203	Involved in neural maturation and synaptogenesis	Triplosensitivity	Developmental and epileptic encephalopathy 2	300672
Xp22.12	*CNKSR2*	connector enhancer of kinase suppressor of Ras 2	300724	Plays a role CNS neuronal postsynaptic density (PSD)	Triplosensitivity	Intellectual developmental disorder, X-linked syndromic, Houge type	301008
Xp22.2	*HCCS*	holocytochrome c synthase	300056	Plays a role in mitochondrial respiratory chain (heme attachment to cytochrome C)	Triplosensitivity	Linear skin defects with multiple congenital anomalies 1	309801
Xp22.2	*MID1*	midline 1	300552	Plays a role in linking cytoskeleton-associated mRNA transport and translation control factors with mTOR gene	Triplosensitivity	Opitz GBBB syndrome	300000
Xp21.2	*NR0B1*	nuclear receptor subfamily 0 group B member 1	300473	Special type of the nuclear receptor (NR) superfamily by acting as a coregulatory protein that inhibits the transcriptional activity of other NRs	Triplosensitivity	46XY sex reversal 2, dosage-sensitive	300018
Xp22.2	*OFD1*	OFD1 centriole and centriolar satellite protein	300170	Plays a role in regulation of microtubule dynamics	Triplosensitivity	Retinitis pigmentosa 23	300424
Xp22.11	*PTCHD1*	patched domain containing 1	300828	Plays a role in hedgehog signaling pathway	Triplosensitivity	{Autism, susceptibility to, X-linked 4}	300830
Xp22.12	*RPS6KA3*	ribosomal protein S6 kinase A3	300075	Plays a role in cell cycle progression, differentiation, and cell survival	Triplosensitivity	Coffin-Lowry syndrome	303600
Xp22.13	*RS1*	retinoschisin 1	300839	Retina cell-cell adhesion and interactions	Triplosensitivity	Retinoschisis	312700
Xp22.11	*SMS*	spermine synthase	300105	Involved in the synthesis of polyamines from arginine and methionine	Triplosensitivity	Intellectual developmental disorder, X-linked syndromic, Snyder-Robinson type	309583
Xp22.2	*AP1S2*	adaptor related protein complex 1 subunit sigma 2	300629	Recruitment of clathrin and sorting signals recognition	Haploinsufficiency	Pettigrew syndrome	304340
Xp22.2	*CLCN4*	chloride voltage-gated channel 4	302910	encodes for voltage-gated chloride channel	Haploinsufficiency	Raynaud-Claes syndrome	300114
Xp22.2	*FANCB*	FA complementation group B	300515	Part of Fanconi anemia core complex	Haploinsufficiency	Fanconi anemia, complementation group B	300514
Xp21.2	*GK*	glycerol kinase	300474	Catalyzes the phosphorylation of glycerol to glycerol-3-phosphate	Haploinsufficiency	Glycerol kinase deficiency	307030
Xp21.3-p21.2	*IL1RAPL1*	interleukin 1 receptor accessory protein like 1	300206	Plays a role in synaptic regulation and regulation	Haploinsufficiency	Intellectual developmental disorder, X-linked 21	300143
Xp22.2	*MSL3*	MSL complex subunit 3	300609	Plays a major role in acetylation of histone H4	Haploinsufficiency	Basilicata-Akhtar syndrome	301032
Xp22.2-p22.13	*NHS*	NHS actin remodeling regulator	300457	Plays a role in actin remodeling and cell morphology	Haploinsufficiency	Cataract 40, X-linked	302200
Xp22.12	*PDHA1*	pyruvate dehydrogenase E1 subunit α 1	300502	Catalyzing the irreversible conversion of pyruvate into acetyl-CoA	Haploinsufficiency	Pyruvate dehydrogenase E1-α deficiency	312170
Xp22.11	*PHEX*	phosphate regulating endopeptidase X-linked	300550	Encodes for an integral membrane zinc-dependent endopeptidase protein	Haploinsufficiency	Hypophosphatemic rickets, X-linked dominant	307800
Xp22.2	*PIGA*	phosphatidylinositol glycan anchor biosynthesis class A	311770	Plays a role in GPI (Glycosylphosphatidylinositol) anchoring biosynthesis	Haploinsufficiency	Multiple congenital anomalies-hypotonia-seizures syndrome 2	300868
Xp22.31	*STS*	steroid sulfatase	300747	Encodes for steroid sulfatase protein that plays a role in estrogen, androgen, and cholesterol synthesis	Haploinsufficiency	Ichthyosis, X-linked	308100
Xp22.2	*TRAPPC2*	trafficking protein particle complex subunit 2	300202	Member of TRAPP complex that plays a role in intracellular vesicle trafficking	Haploinsufficiency	Spondyloepiphyseal dysplasia tarda	313400

**Table 2:** The large duplications (of more than 1 Mb) on the short arm of the X chromosome that have been previously reported in patients are listed, along with their associated symptoms; data related to our patient are included for comparison.

Year	Citation	Patient	Location	Size	Genes	Clinical Manifestations
2006	31	1	ChrX:9,700,000–16,400,000	7 MB	*MID1*, *ARHGAP6*, *MSL3L1*	Mental Retardation
						Facial dysmorphism
						Hearing loss/Presbycusis
						Pectus excavatum
						Arachnodactyly
						Atrophy of interdigital muscles
2011	33	2	ChrX:9,750,000–18,710,000	9 MB	59 genes of which seven are involved in syndromic X-linked intellectual deficiency, namely *MID1*, *HCCS*, *OFD1*, *FANCB*, *AP1S2*, *CDKL5* and *NHS.*	Mental retardation
						Developmental delay
						Dysmorphic features
						Facial dysmorphism (hypertelorism, broad nasal bridge, widow’s peak)
						Genitourinary abnormalities
						Heart defects
						Short stature
						Scoliosis
						Hypertelorism
						Neurodevelopmental disorders
						Diaphragmatic hernia
2022	29	3	ChrX:19,563,240–20,597,641	1 MB	*SH3KBP1*, *EIF1AX* and *RPS6KA3*	Delayed speech
						Language development delay
						Seizure
						Joint laxity
		4	ChrX: 19,825,290–20,930,431	1.1 MB	*SH3KBP1*, *EIF1AX* and *RPS6KA3*	Specific learning disability
		5	ChrX: 19,651,193–20,700,691	1.05 MB	*SH3KBP1*, *EIF1AX* and *RPS6KA3*	Intellectual disability
2008	30	6	ChrX: 1,5000,000–23,500,000	8.5 MB	*CDKL5*, *RPS6KA3*	Facial dysmorphism
						Bilateral inguinal hernia
						Downslanted palpebral fissures
						Upslanted palpebral fissures Bilateral inguinal hernia
						Epilepsy
						Brain tumor at age 3 yrs
2022	27	7	ChrX: 24,513,979–27,864,451	3.35 MB	*PDK3*, *PCYT1B*, *POLA1*, *SCARNA23*, *ARX*, *MAGEB18*, *MAGEB6B*, *MAGEB6*, *MAGEB5*, *PPP4R3C*, *DCAF8L2* and *MAGEB10*	Autism
		8	ChrX: 24,810,754–27,125,219	2.3 MB	*POLA1*, *ARX*, *MAGEB18*, *MAGEB6B*, *MAGEB6* and *MAGEB5*	Intellectual disabilities
						Short stature
Current case	4	9	ChrX: 7,137,718–30,739,112	23.6 MB	Around 143 genes, of which 11 are triplosensitive: *ANOS1*, *ARX*, *CDKL5*, *CNKSR2*, *HCCS*, *MID1*, *NR0B1*, *OFD1*, *PTCHD1*, *RPS6KA3*, *RS1*, *SMS*, *AP1S2*, *CLCN4*, *FANCB*, *GK*, *IL1RAPL1*, *MSL3*, *NHS*, *PDHA1*, *PHEX*, *PIGA*, *STS*, *TRAPPC2*.	Neurodevelopmental delay
						Intellectual disability
						Psychomoter delay
						Hypotonia
						Failure to thrive
						CNS malformation (abnormal T2 signals in the basal ganglia, thalami, and brainstem, atrophy of the hippocampus
						Seizures
						Strabismus
						Facial dysmorphism
						Short stature
						Scoliosis
						Joint hypermobility/laxity,
						Optic nerve hypoplasia
						Feeding difficulty
						GE reflux

## Data Availability

All data are available from the corresponding author upon reasonable request.
